# Understanding the influence of geometry and material properties on femoral and tibial stress and strain analysis in a paediatric population for single-leg standing simulation

**DOI:** 10.3389/fbioe.2026.1752875

**Published:** 2026-06-11

**Authors:** Seraina Kämpf, Yidan Xu, Julie Choisne

**Affiliations:** 1 Institute for Biomechanics, ETH Zurich, Zürich, Switzerland; 2 Auckland Bioengineering Institute, The University of Auckland, Auckland, New Zealand

**Keywords:** femur, finite element analysis, paediatric bone, statistical shape and density model, tibia

## Abstract

**Introduction:**

Paediatric Finite Element (FE) models of bones are scarce due to data Q7 unavailability. Recent research has developed statistical shape and density models (SSDMs) for paediatric populations, enabling good predictions of bone geometry and bone mineral density (BMD). However, the impact of bone shape and material property accuracy on stress and strain calculation remains poorly understood. Therefore, the aim of this study was to investigate the influence of geometry and material property accuracies on Von Mises stress and 1st principalstrain distribution in paediatric femora and tibiae.

**Methods:**

Patient-specific bone geometry and BMD were extracted from 330 paediatric CT scans. Subsequently, two paediatric SSDMs were created, which can predict bone shape and BMD for the femur and tibia, respectively. Next, nine FE models were built for each femur and tibia in the dataset using different inputs for geometry and material properties: i) patient-specific from the CT scans; ii) predicted from the SSDM; iii) generic from the literature. Von Mises stresses and 1st principal strains were compared between the models under identical loading conditions.

**Results:**

The results showed that using SSDM-predicted or generic material properties increased the Von Mises stress and 1st principal strain Root Mean Square Errors compared to using material properties derived from CT scans. SSDM-predicted geometry and genericbased geometry associated with CT-based material properties showed the lowest errors of the eight models compared to the CT-based geometry and material properties model.

**Discussion:**

The results show a trend toward a greater influence of material property inaccuracies on the Von Mises stress and 1st principal strain distribution than geometry for this paediatric population.

## 1 Introduction

Paediatric bone structure and function are poorly understood (Kumar et al., 2023), despite a strong interest in a better comprehension and prediction of mechanical loading conditions with respect to trauma, movement disorders and surgical interventions (Bavil et al., 2025; Szabo and Rimnac, 2022). Finite Element (FE) modelling is used frequently in adults to study bone fracture risk (Keyak et al., 2011; Johannesdottir et al., 2018; Falcinelli et al., 2014; Qasim et al., 2016), bone mechano-adaptation (Meslier and Shefelbine, 2023), joint contact mechanics (Kakavand et al., 2024) and surgical procedures (Yunus et al., 2022). In contrast, subject-specific paediatric FE models are scarce due to the unavailability of imaging data required for model development. One common way to build paediatric computational models is to scale a generic adult template (Carriero et al., 2011; Delp et al., 2007). However, using adult generic data for children leads to inaccurate results, as paediatric bone geometry and function differ from those of adults (Carman et al., 2022). Bones develop on a heterogeneous level (Altai et al., 2018). During childhood and continuing into early adulthood, the bones experience pronounced changes in their size, morphology, and internal organisation (Goldman et al., 2009). Recent investigations have demonstrated an age-related increase in volumetric BMD in the femur and tibia, with the most pronounced changes observed in the diaphyseal regions (Choisne et al., 2025). Using subject-specific geometry and bone mineral density from medical imaging data to build paediatric models overcomes the disadvantage of inaccurate representations of generic models. However, medical imaging, such as MRI, is expensive and does not include information on bone mineral density (Davico et al., 2020). On the other hand, X-rays and DEXA are low-cost but can only give 2-dimensional information, lacking volumetric information. CT images would be the gold standard; however, it would involve radiation, which poses significant cancer risks, with the paediatric population being particularly sensitive (Pearce et al., 2012; Brenner et al., 2001). Therefore, current paediatric computational models are based on small cohorts (n = 1–3) (Castro et al., 2019; Incze-Bartha et al., 2023; Kainz et al., 2020; Yadav et al., 2016; Yadav et al., 2017) or a limited field of view (Castoldi et al., 2024).

Recently, researchers have developed statistical shape and density models (SSDMs) for both adult (Nico et al., 2012; Grassi et al., 2014; Nolte and Bull, 2019; Steiner et al., 2021) and paediatric (Castoldi et al., 2024; Xu et al., 2025a; Xu et al., 2025b) populations, enabling predictions of bone geometry and BMD based on sparse input data. While such studies quantitatively describe geometry and BMD prediction errors, the impact of bone shape and material property accuracies on stress and strain prediction remains poorly understood. From a dataset of two adult femora and one paediatric femur, errors in geometry were found to be the dominant variable influencing stresses (Taddei et al., 2006). But in general, the influence of geometric and material assignment errors on finite element results is not predictable beforehand, and the results seem to be dependent on the subject (Taddei et al., 2006).

Therefore, the goal of this study was to investigate the influence of geometry and material properties on Von Mises stress and principal strain results in paediatric femora and tibiae. More precisely, we explored how SSDM-based and generic representations of geometry and material properties affect stress and strain relative to the gold standard (CT-based). This was done for the whole bone and for different regions of interest (ROIs) within the bone. Additionally, we investigated which of the two modalities, geometry or material properties, had the greater influence on Von Mises stress and Principal strain errors.

## 2 Methods

### 2.1 Data acquisition and data extraction

Post-mortem CT scans were collected by the Victorian Institute of Forensic Medicine (VIFM, Melbourne, Australia) between 2006 and 2019. The dataset used in this study consisted of full body CT scans of 330 children (136 female, 12 ± 5 years old [4–18], height: 148 ± 24 cm [96–192], weight: 49 ± 22 kg [14–140] (Supplementary Figure SA). Ethical approval was obtained from the VIFM Ethics Committee (2023-Choisne-1143–2385/3) and the Auckland Health Research Ethics Committee at the University of Auckland (AH24671). This study used retrospective data, which were collected by the VIFM for autopsy purposes. Before autopsy, the VIFM obtained written consent from the individual’s legal guardian. All methods were performed in accordance with the relevant guidelines and regulations.

A calibration phantom (Model 3 CT Calibration Phantom, Mindways Inc. Phantom, Mindways Inc.) was included in the scans. Slice thickness ranged from 0.5 to 2 mm, and pixel spacing varied between 0.57 × 0.57 mm and 1.27 × 1.27 mm. The matrix size was 512x512 for all CT scans and the maximum field of view was 500 mm. Former work reported the data extraction (Carman et al., 2022; Xu et al., 2025a). In summary, from the 330 full-body CT scans, the left and right femora and tibiae were segmented and reconstructed (examples of segmentation by year range can be found in the Supplementary Table SF). Subsequently, after reconstructing the left and right bones, the left-sided bones were mirrored to appear right-sided, thereby increasing the sample size, as the statistical shape and density model was built only to produce right-sided bone predictions. Children with conditions affecting the lower limb structure or function were previously excluded from the dataset. Some bones were not fully visible in the CT scans and were therefore not segmented or included in the analysis. In the end, the total number of segmented and reconstructed femora was 657 (329 right femora and 328 left femora), and the total number of tibiae was 652 (326 right tibiae and 326 left tibiae). The number of unique femora and tibiae used in this study, distributed by age and sex, can be found in the Supplementary Figure SA; Supplementary Table SA. Surface and volumetric mesh registration were reported in previous studies (Carman et al., 2022; Xu et al., 2025a). For both the femur and tibia, a template surface mesh (F, 5Y, 111 cm) was built from one individual and fitted to the rest of the dataset to ensure nodal correspondence. Based on this surface mesh, a 4-node tetrahedral template mesh with an element size of 2 mm was produced using TetGen (Si, 2015). A convergence study was conducted for the template meshes and their respective largest meshes (M, 17Y, 192 cm) using single leg standing boundary conditions (§2.3.3). Convergence was defined as the point at which the average Von Mises stress change was less than 1% upon further mesh refinement. The resulting volumetric template mesh consisted of 21,900 nodes and 122,964 elements for the femur and 25,874 nodes and 150,164 elements for the tibia. The meshes for both femur and tibia were then morphed separately to the rest of their dataset using a host mesh fitting approach (Xu et al., 2025a; Fernandez et al., 2004). The average element size (average element edge length) was 3.1 ± 0.65 mm across all bones. Due to the common topology, the element size scaled with growth, with an R^2^ = 0.82 between element size and age. A comprehensive analysis of the mesh-to-pixel resolution ratio is provided in Supplementary Table SB.

BMD was extracted using Bonemat (Rolling Version, Istituto Ortopedico Rizzoli, Bologna, Italy) (Taddei et al., 2007) based on a Hounsfield units-BMD relationship from the calibration phantom present in the CT scan (Choisne et al., 2025).

### 2.2 Statistical shape and density Model

The methodology for building SSDMs was previously reported (Xu et al., 2025a; Xu et al., 2025b). In short, femur and tibia SSDMs were constructed from the CT-derived subject-specific volumetric models of paediatrics described in §2.1. Variations in bone shape and densities were captured for the femur and tibia using principal component analysis (PCA). The femur PCA was built independently of the tibia PCA. Principal components (PC) weights and mean shapes were obtained from the PCA. For the leave-one-out analysis, left and right bones of the target participant were excluded from the SSDM training set. Partial least squares regression was then used to predict the PC weights for this participant based on demographic characteristics (age, height, weight, and sex) and bone linear measurements (epicondylar width and femoral length for the femur, and condylar width, malleolar width, and tibial length for the tibia). The predicted PC weights and the mean mesh were used to reconstruct the shape and density for each participant’s bones.

### 2.3 Generation of finite element models

Three different approaches were used to represent the geometry and material properties in the FE models:

- CT-based: geometry obtained from CT-derived subject-specific meshes, with bone mineral density (BMD) extracted from CT and subsequently converted to material properties.
- SSDM-based: geometry and BMD predicted from the SSDM in a leave-one-out analysis; SSDM-based material properties were then assigned by converting the SSDM-predicted BMD using the same density-elasticity relationships as for CT-based models.
- Generic (Gen): geometry obtained from the isometrically scaled mean SSDM shape; BMD and material properties assigned from literature-based generic values.

Using all combinations, a total of 9 FE models were built for each of the 657 femora and 652 tibiae. The resulting models, along with their names, are presented in Table 1. The geometry is named first, followed by material properties (e.g., CT-SSDM: CT-based geometry, SSDM-based material properties).

**TABLE 1:** Naming of the nine different FE models, geometry is named first, followed by material properties.

Geometry	Material properties		
	CT	SSDM	Gen
CT	CT-CT	CT-SSDM	CT-Gen
SSDM	SSDM-CT	SSDM-SSDM	SSDM-Gen
Gen	Gen-CT	Gen-SSDM	Gen-Gen

The following paragraphs describe the three different approaches to modelling the geometry (§2.3.1, Geometry) and material properties (§2.3.2, Material Properties). All simulations were performed in FEBio 4.3.0 (Febio Software Suite, United States) with a quasi-static analysis (Maas et al., 2012). Specific ROIs within the femur (head, neck, trochanter, shaft and distal end) and tibia (proximal end, shaft, distal end) were identified for a detailed analysis at a later stage (Xu et al., 2025a). For both bones, the proximal end is defined as the upper 20% and the distal end as the lower 20% of the total length. (Gosman et al., 2013).

#### 2.3.1 Geometry

The CT-based geometry was the segmented bone shape from the CT images. The SSDM-based geometry was obtained directly from the predicted shape in the leave-one-out analysis (Xu et al., 2025a; Xu et al., 2025b). The generic geometry was the resulting average bone shape from the PCA, which was scaled isometrically using the individual bone length (femoral length and tibial length, respectively) from the CT scan. The FE mesh for all modelling approaches was a fixed-topology 4-node tetrahedral mesh generated within the SSDM framework (Steiner et al., 2021). The resulting volumetric mesh consisted of 21,900 nodes and 122,964 elements for the femur and 25,874 nodes and 150,164 elements for the tibia.

#### 2.3.2 Material properties

The Poisson’s ratio was set to 0.3 for all the models (Querol et al., 2006). For the CT-based material properties, the BMD was extracted from the CT scans using the calibration phantom (Xu et al., 2025a). Then, the BMD was transformed to Young’s Modulus (*E*) values based on the following equations. Ash, apparent density and CT density are all expressed in *g/cm* ^*3*^, *E* is expressed in *MPa*.

Femur: The equations for calculating ash and apparent density from CT density are reported by Schileo et al. (2008).

$$\varphi_{ash}=0.8772\varphi_{CT}+0.07895$$ (1)

$$\varphi_{app}=\frac{1}{0.6}\varphi_{ash}$$ (2)

The Equations 1, 2 were derived from adult bones. The Young’s modulus for each element was estimated using the well-established relationship by Morgan et al. (Morgan et al., 2003)

$$E_{Femur}=6850\rho_{app}^{1.49}=14664{\left(0.8772\varphi_{CT}+0.07895\right)}^{1.49}$$ (3)

Equation 3 is based on femoral neck specimens of adult human bone. It was chosen because it is recommended to use relationships derived from the same anatomical site on which the FE investigation is planned (Helgason et al., 2008). Due to the scarcity of paediatric data, extrapolating adult density-elastic relationship for paediatric is a common approach. Currey et al. (1996) conducted a series of fracture (proximal femurs) and impact (distal femurs) experiments using specimens between 4 and 82 years old, and found that the age and ash density were highly correlated (*R* ^2^ = 0.74). This is in agreement with a study conducted by Öhman et al. (2011), which showed a strong correlation between ash density and compressive elastic (orYoung’s) modulus (*R* ^2^ = 0.86–0.91) (4–15 years old). The study suggested that the methods developed for modelling adult bones could be tailored to paediatric applications.

Tibia: The equations to calculate the ash density from the CT density, as well as the relationship between CT density and elasticity, are reported by Keyak et al. (1994).

$$\varphi_{ash}=1.06\varphi_{CT}+0.0389$$ (4)

$$E_{Tibia}=11300{\rho_{ash}}^{1.9}=11300{\left(1.06\varphi_{CT}+0.0389\right)}^{1.9}$$ (5)

The Equations 4, 5 were derived from adult proximal tibiae (Keyak et al., 1994).

The SSDM-based material properties used the predicted BMD from the leave-one-out analysis and transformed it to Young’s moduli with the same equations as for the CT material properties: Equations 1–3 for the femur and Equations 4, 5 for the tibia.

For the generic material properties, we chose to differentiate between trabecular bone, cortical bone and bone marrow. To identify the regions associated with the three densities, we examined the CT scans of the heaviest and lightest children to determine a suitable threshold for differentiating between the three densities. The suitable CT thresholds found were set to 650 Hounsfield Units (HU) (threshold_cortical_) and 150 HU (threshold_trabecular_). We performed quality insurance by randomly selecting 20 individuals and checking that the threshold identified would be able to identify cortical bone, trabecular bone and bone marrow. For each femur and tibia, the cortical bone, trabecular bone and bone marrow were identified by selecting the elements with bone mineral densities such as; cortical bone was assigned if density ≥ threshold_cortical_, bone marrow was assigned if density < threshold_trabecular_, and trabecular bone was assigned otherwise.

The Young’s modulus values for cortical bone, trabecular bone and bone marrow in the generic models were set according to Kainz et al., (2020): E_cortical_ = 20,000 MPa, E_trabecular_ = 600 MPa, E_marrow_ = 1 MPa.

As paediatric data is scarce, this was the only study we found using generic material properties assigned to paediatric bones for FEA. These values are aligned with previous studies on cortical bone elastic modulus (Szabo and Rimnac, 2022).

#### 2.3.3 Boundary conditions

All femora and tibiae were aligned according to the recommendations of the International Society of Biomechanics coordinate system (Xu et al., 2025a; Wu et al., 2002). The boundary conditions modelled single-leg standing and stayed the same for all models.

Femur: A hip joint contact force (JCF) equal to 2.4 times body weight was applied to the outer hemispherical surface of the femoral head (Hölzer et al., 2013). This value represents the average peak contact force measured in the hip using instrumented implants during single-leg standing (Bergmann et al., 2010). The force was applied at angles α = 84.3°, β = 1.7°, and γ = 30°. Nodes on the contact surface were constrained to allow movement only along the superior-inferior axis, while nodes at the distal end of the femur were fully constrained in all degrees of freedom.

Tibia: A knee JCF equal to 2.5 times body weight was applied to the tibial plateau. This value corresponds to the average peak contact force recorded in the knee using instrumented implants during single-leg standing (Kutzner et al., 2010). Nodes on the tibial plateau were constrained to permit movement only along the superior-inferior axis, while the distal end of the tibia was fully fixed in all degrees of freedom (Xu et al., 2025a; Xu et al., 2025b).

### 2.4 Evaluation of finite element models

To evaluate the performance of the models, three different measures were investigated for the femora and tibiae: Von Mises (effective) stress and first principal strain, mean values, root mean square error (RMSE) and coefficient of determination (*R* ^2^). The 1st principal strain was selected as the primary strain metric to represent the maximum tensile environment. This choice was based on prior analyses using this FE pipeline, which indicated that error trends between CT-derived gold standards and predicted models are consistent across both tensile (1st) and compressive (3rd) principal strains in paediatric long bones (Fratzl-Zelman et al., 2009). To ensure clarity and avoid data redundancy, only the first principal strain (maximum tensile strain within each element) and von Mises stress are reported as indicators of the mechanical response.

#### 2.4.1 Mean values

The mean values with standard deviation (SD) of the Von Mises stress and first principal strain for each segmented bone and each model were determined. To assess whether specific ROIs are more sensitive to changes in the material properties or geometry, mean values with SD were computed for each ROI as well.

Statistical analysis: The mean values were analysed using SPSS Statistics for Windows, version 30.0 (IBM Corp., Armonk, N.Y., United States). To look at the influence of the geometry and material properties separately, two Friedman tests (non-parametric equivalent to repeated measures ANOVA as the dataset was not normally distributed) were performed for each bone and each ROI. To evaluate the influence of material properties, the test was performed on models with CT-based geometry and varied material properties (CT-CT vs. CT-SSDM vs. CT-Gen). To evaluate the impact of geometry, another test was performed for models with CT-based material properties and varied geometry (CT-CT vs. SSDM-CT vs. Gen-CT). This procedure was chosen because geometry and material properties were coupled in the SSDM and, therefore, not independent. The significance level was set at α = 0.05. When a statistically significant difference was observed between the groups, the Wilcoxon signed-rank test assumption of a symmetrical distribution of paired differences was visually assessed using boxplots. Post-hoc tests with Bonferroni correction were then performed, yielding a corrected significance level of α_new_ = 0.017.

#### 2.4.2 Root mean square error

To answer the question of which modality, geometry, or material properties have the biggest influence on the stress and strain error, the RMSE of von Mises stress and first principal strain was calculated for each model and each ROI based on an element-to-element comparison with the CT-CT model, which served as the gold standard. To better understand the resulting errors, the RMSE for geometry (surface node distance error) and material properties (Young’s modulus) were calculated for each ROI using both the predicted (SSDM) and generic approach, with respect to the CT-CT model as gold standard.

#### 2.4.3 Determination coefficient

To evaluate model performance, the determination coefficient (*R* ^2^) was evaluated between the predicted and CT-CT models using element-wise distributions of von Mises stress and first principal strain to quantify model agreement. The CT-CT model served as the reference for all comparisons. *R* ^2^ values were calculated for each bone and each model.

Negative *R* ^2^ values indicate that the tested model predicts stress or strain distributions less accurately than a reference model using the mean of the CT-CT distribution, and therefore reflect performance worse than a mean - value predictor.

## 3 Results

### 3.1 Mean values

The mean stress and strain values ±SD for the whole femur and tibia for each model are depicted in Table 2. The mean stress (Supplementary Table SC1) and mean strain (Supplementary Table SC2) values ±SD for each the femoral and tibial ROIs are shown in the Supplementary Material. The results from the Friedman tests (Supplementary Table SD) and the post-hoc analysis (Supplementary Table SE) are also included in the Supplementary Material.

**TABLE 2:** Femoral and tibial whole bone Von Mises stress and first principal strain mean values ±SD.

​	Mean values ±SD			
	Femur		Tibia	
Model	Von mises stress	1st principal strain	Von mises stress	1st principal strain
CT-CT	1.49 ± 0.34	0.16 ± 0.04	1.81 ± 0.42	0.28 ± 0.08
CT-SSDM	1.47 ± 0.33*	0.16 ± 0.03*	1.76 ± 0.40*	0.28 ± 0.06*
CT-Gen	1.63 ± 0.39*, †	0.39 ± 0.08*, †	1.79 ± 0.42*, †	0.34 ± 0.07*, †
SSDM-CT	1.47 ± 0.31*	0.16 ± 0.04*	1.79 ± 0.37	0.28 ± 0.07
SSDM-SSDM	1.45 ± 0.31	0.16 ± 0.03	1.74 ± 0.36	0.28 ± 0.05
SSDM-Gen	1.60 ± 0.35	0.38 ± 0.07	1.76 ± 0.36	0.33 ± 0.06
Gen-CT	1.50 ± 0.36	0.17 ± 0.04	1.82 ± 0.45	0.28 ± 0.08
Gen-SSDM	1.47 ± 0.35	0.16 ± 0.04	1.77 ± 0.44	0.28 ± 0.06
Gen-Gen	1.63 ± 0.40	0.39 ± 0.09	1.78 ± 0.44	0.34 ± 0.08

Stress values are in [MPa], strain values in [mm/m]. * Denotes statistical difference compared with CT-CT and † denotes statistical difference compared to CT-SSDM (p < 0.05). Note that only CT-CT vs. CT-SSDM vs. CT-Gen and CT-CT vs. SSDM-CT vs. Gen-CT were included in the statistical analysis.

#### 3.1.1 Von Mises Stress

Whole bone: Both bones showed a statistically significant difference in mean values depending on material properties (Table 2; Supplementary Table SD in Supplementary Material). The post-hoc analysis revealed that all models statistically differed from one another in terms of Von Mises stress output (Supplementary Table SE).

For the geometry, the femoral models showed statistically significantly different mean stress values, but not for the tibial models (Table 2; Supplementary Table SD). Post-hoc analysis in the femur revealed a statistically significant difference only between CT-CT vs. SSDM-CT (Supplementary Table SE).

Regions: All regions from either the femur or tibia exhibited a statistical difference in mean stress values based on material properties, whereas only a few femoral regions showed a statistically significant difference based on geometry (Supplementary Tables SC1, SD). In the post-hoc analysis, statistically significant differences were found in models with varying material properties for every comparison, except for CT-CT vs. CT-SSDM in the distal femur. For varying geometries, the only statistically significant difference was found in the trochanter and femoral shaft between CT-CT and SSDM-CT (Supplementary Table SE).

#### 3.1.2 First principal strain

Whole bone: In the femur, models with generic material properties showed strain values more than twice as high as those of the other models (Table 2). In the tibia, the models with generic material properties also showed higher values, but the differences were smaller than those in the femur. Both bones showed a statistically significant difference in mean strains depending on material properties (Supplementary Table SD). A statistically significant difference in mean strains depending on the geometry was found in the femur but not in the tibia (Supplementary Table SD). Post-hoc analysis revealed that the comparison between CT-CT vs. CT-SSDM was statistically non-significant in the femur and tibia, while the comparison between CT-CT vs. SSDM-CT in the femur was found to be significantly different (Supplementary Table SE).

Regions: A statistically significant difference in mean strains was observed depending on the material properties in all regions (Supplementary Tables SC2, SD, Supplementary Material). Post-hoc analysis showed that in general, the three models with CT-based geometry and varying material properties were statistically significantly different from one another (Supplementary Table E). A few exceptions occurred between CT-CT vs. CT-SSDM in the femoral shaft, distal femur and proximal and distal tibia. There was a statistically significant difference depending on geometry in the femoral neck, femoral shaft, distal femur and proximal tibia (Supplementary Table SD). Post-hoc analysis revealed a statistically significant difference in the femoral shaft and distal femur between CT-CT vs. SSDM-CT and between SSDM-CT vs. Gen-CT (Supplementary Table SE). In the proximal tibia, a statistically significant difference was only observed between SSDM-CT vs. Gen-CT.

### 3.2 Root mean square error

#### 3.2.1 Von Mises Stress RMSE

**FIGURE 1:**
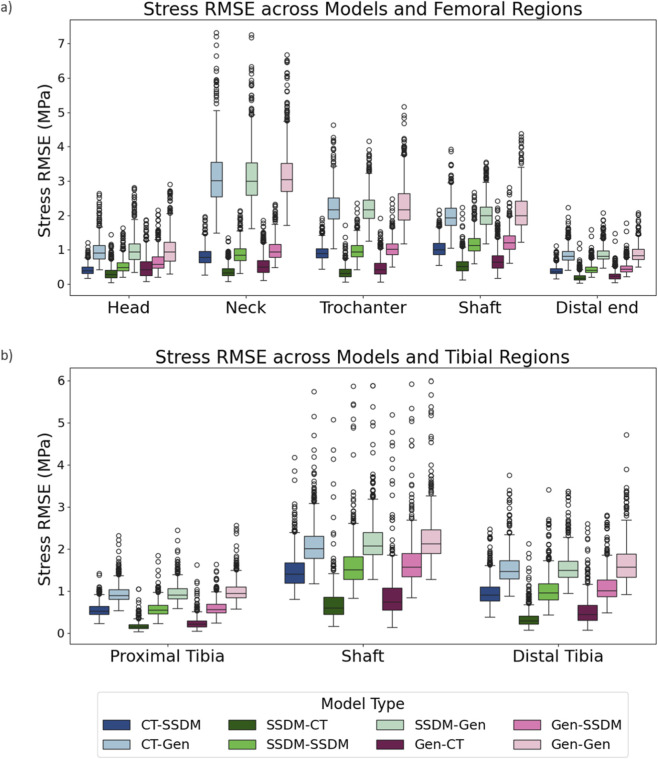
Von Mises Stress RMSE for each model across **(a)** femoral and **(b)** tibial regions of interests compared to the subject-specific geometry and material properties (CT-CT).

The models with generic material properties showed higher stress RMSE than models with CT- or SSDM-based material properties in the whole bone and in all ROIs in the femur (Figure 1a; Supplementary Figure SB1) and tibia (Figure 1b; Supplementary Figure SB2). Moreover, models with CT-based material properties showed the lowest stress errors in all ROIs.

#### 3.2.2 First principal strain RMSE

**FIGURE 2:**
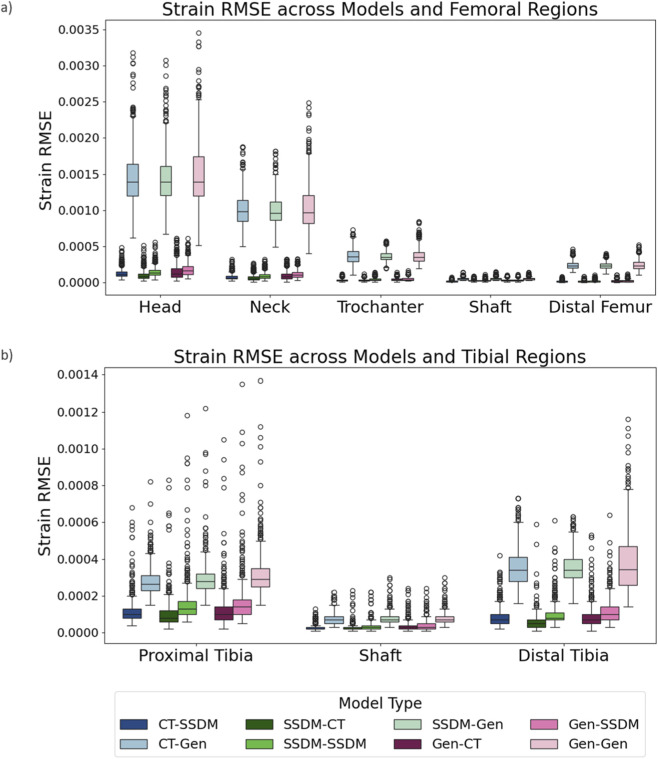
First principal strain RMSE for each model across **(a)** femoral and **(b)** tibial regions of interests compared to the subject-specific geometry and material properties (CT-CT).

The models with generic material properties showed higher strain errors than models with CT- or SSDM-based material properties in the whole bone and ROIs in the femur (Figure 2a; Supplementary Figure SC1) and tibia (Figure 2b; Supplementary Figure SC2). Differences between the models with CT- or SSDM-based material properties were not clearly distinguishable in terms of strain errors.

#### 3.2.3 Geometry and material properties RMSE

**FIGURE 3:**
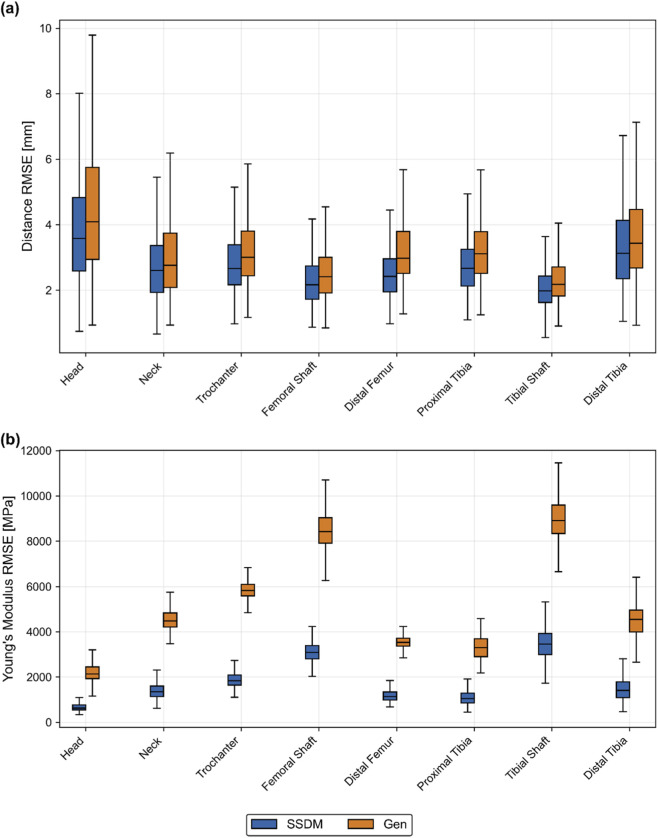
Regional **(a)** distance (mm) and **(b)** Young’s modulus (MPa) RMSE for both predicted (SSDM) and generic geometry and material properties in all femoral and tibial ROIs. The CT-based geometry and material properties served as the gold standard.

The SSDM-predicted geometry showed low surface errors in all femoral and tibial ROIs (Figure 3a). The generic geometry showed higher surface errors than the SSDM-predicted geometry in all ROIs and higher surface errors in tibial ROIs than in femoral ROIs.

The generic material properties showed higher Young’s modulus RMSE than the SSDM-predicted material properties in all ROIs (Figure 3b). The highest errors were observed within each modelling type in the femoral and tibial shafts.

### 3.3 Determination coefficient

The mean coefficient of determination (*R* ^2^) for each model across all participants are depicted in Table 3. The models with CT-based material properties exhibited the highest *R* ^2^ values, followed by those with SSDM-based material properties. The models with generic material properties showed the lowest coefficients and performed worse in the strain calculation compared to the stress calculation. The best possible score is 1, while negative coefficients mean that the model performs arbitrarily worse.

**TABLE 3:** Average coefficient of determination (*R* ^2^) ± SD in stress and strain between the CT-CT model compared to the other models.

​	Mean determination coefficient (R2) ± SD			
​	Femur		Tibia	
Model	Von mises stress	1st principal strain	Von mises stress	1st principal strain
CT-SSDM	0.86 ± 0.04	0.95 ± 0.04	0.80 ± 0.06	0.82 ± 0.17
CT-Gen	0.33 ± 0.09	−5.00 ± 2.21	0.57 ± 0.07	−0.63 ± 1.08
SSDM-CT	0.96 ± 0.03	0.96 ± 0.08	0.96 ± 0.04	0.88 ± 0.15
SSDM-SSDM	0.83 ± 0.05	0.93 ± 0.05	0.77 ± 0.07	0.70 ± 0.31
SSDM-Gen	0.31 ± 0.16	−5.01 ± 2.43	0.54 ± 0.10	−0.72 ± 1.19
Gen-CT	0.93 ± 0.09	0.91 ± 0.14	0.92 ± 0.11	0.81 ± 0.21
Gen-SSDM	0.80 ± 0.08	0.89 ± 0.12	0.74 ± 0.10	0.65 ± 0.35
Gen-Gen	0.22 ± 0.40	−5.77 ± 4.18	0.49 ± 0.18	−1.04 ± 1.62

#### 3.3.1 Von Mises Stress

**FIGURE 4:**
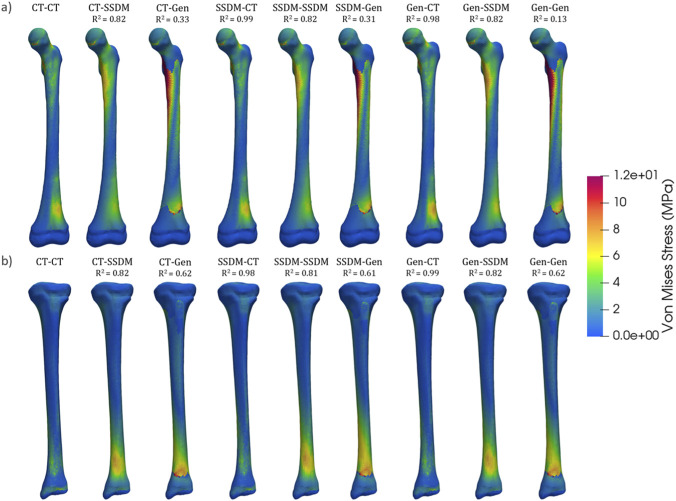
Femoral **(a)** and tibial **(b)** models with corresponding determination coefficients (*R* ^2^) for the participant with the highest *R* ^2^ value for von Mises stress from the respective dataset. Participant **(a)** F, 5 years, H: 118 cm, W: 25 kg, highest *R* ^2^ in the SSDM-CT model (0.99); Participant **(b)** M, 17 years, H: 167 cm, W: 47 kg, highest *R* ^2^ in the Gen-CT model (0.99).

**FIGURE 5:**
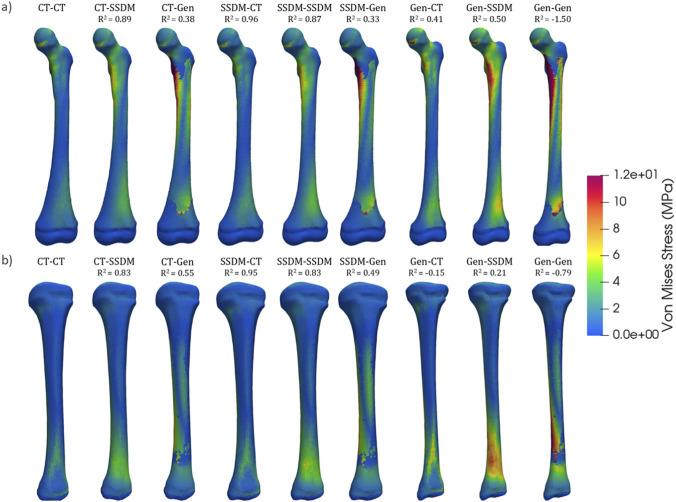
Femoral **(a)** and tibial **(b)** models with corresponding determination coefficients (*R* ^2^) for the participants with the lowest *R* ^2^ value for Von Mises stress from the respective dataset. Participant **(a)** M, 4 years, H: 110 cm, W: 20 kg, lowest *R* ^2^ value in the Gen-Gen model (−1.50); Participant **(b)** M, 5 years, H: 116 cm, W: 27 kg, lowest *R* ^2^ value in the Gen-Gen model (−0.79).

Models with generic material properties showed higher stresses in the region of the lesser trochanter and along the shaft in the femur (Figures 4a, 5a) and at the lower shaft and the distal end in the tibia (Figures 4b, 5b) compared to the CT-CT stress distribution. In the models with generic material properties, a clear boundary was apparent between the proximal end of the femur and the femoral shaft, with a similar pattern between the shaft and the distal end in both the femur and tibia. The stress pattern was similar in models with CT-based material properties compared to the CT-CT model in both bones. In the tibia, models with SSDM-based material properties showed higher stress values in the distal part compared to the CT-CT model.

#### 3.3.2 First principal strain

In the femur, models with generic material properties showed high strains in the region of the femoral head and neck. In both bones, there was a clear distinction between the shaft and the distal end, showing high strains just below that boundary (Supplementary Figures SD, SE).

## 4 Discussion

Nine Finite Element models were developed using a combination of CT-based, SSDM-based, and generic inputs to represent the geometry and material properties of paediatric femora and tibiae. These models were used to investigate the influence of geometry and material properties on stress and strain behaviour in paediatric bones. Overall, in our dataset and under the standardized single-leg-standing condition, changing material assignment from CT-based to SSDM-based to Generic produced larger influence on magnitude-based outcomes (whole-bone/ROI means and element-wise RMSE) and more pronounced decreases in element-wise *R* ^2^ than changing geometry alone. By contrast, while geometry differences have a smaller impact on global *R* ^2^, they were most visible as local pattern errors within ROIs.

### 4.1 Influence of geometry

When looking at the stress in models with CT-based material properties and varying geometry input, most of the femoral and tibial regions did not show statistical differences except for the femoral trochanter and femoral shaft regions between the CT-based and SSDM-predicted geometry. No statistically significant differences in stresses were found between the CT-based and generic scaled geometries. The SSDM-based geometry was generated by adjusting the PC weights to capture individual anatomical variations, whereas the generic geometry scaled the average dataset geometry linearly according to bone length. Thus, as per statistics, the geometry from the generic average model was shaped appropriately for most of the dataset. The generic geometry preserved key global features of the bone, which might be varied by the SSDM prediction. These shape variations (e.g., femoral trochanter and diaphyseal curvature) could have a pronounced effect on local stress distribution. However, the generic geometry cannot account for subject-specific differences and therefore would do worse in the extreme cases. In the tibia, the only statistically significant difference related to varying geometry was observed for the strains in the proximal tibia between the SSDM-predicted and the generic scaled geometry. This could be influenced by the convex shape of the tibial plateau in the young tibiae compared to the more concave tibial plateau in the generic tibia shape, indicating that personalised geometry is important in some regions.

Generally, both stress and strain errors tended to increase, while the coefficient of determination decreased slightly when changing from CT-to SSDM-based geometry. The same observations were seen again when changing from SSDM-based to generic geometry. Although the average stress and strain distribution did not show statistical differences, a closer examination of the RMSE element by element revealed that the generic geometry carried more stress and strain errors than the SSDM-based geometry (Figures 1, 2). This was verified by looking at the distance RMSE between the CT segmentation and the predicted geometry from the SSDM and the scaled generic bones (Figure 3).

### 4.2 Influence of material properties

Statistical analysis revealed that the mean stress and strain comparisons between models with CT-based geometry and varying material properties were statistically significantly different in all regions of the femur and tibia. The post hoc analysis showed a statistically significant difference between CT-CT, CT-SSDM and CT-Gen in almost all ROIs. No statistical differences in stress were found between CT-SSDM and CT-Gen in the femoral neck. No statistical differences were found between CT-CT and CT-SSDM, at the distal femur for stress and strain and at the proximal and distal tibia for strain. It must be considered that only mean values were compared, meaning that underestimation of stresses and strains in some elements and overestimation in other elements in the same ROI do not necessarily influence the mean value. This was most probably the case in the femoral neck. In this region, the stress errors from models with generic material properties were clearly higher than in models with CT- or SSDM-based material properties (Figure 1). The lowest mean strains were observed in the femoral and tibial shafts. A high Young’s modulus leads to a rigid behaviour of the bone, less deformation and therefore small strains and high stresses. In the models with generic material properties, the Young’s modulus in the cortical bone in the shaft was most likely too high for some of our population and not well distributed compared to CT densities, and therefore the observed strains were lower and the stresses higher than in models with material properties from CT or SSDM. In the proximal and distal regions of the bones, the models with generic material properties displayed very high strains compared to the models with CT- and SSDM-based material properties. Particularly in the femur, the models with generic material properties exhibited strains that were twice or three times higher than those of the other models (Supplementary Table SC2). This is consistent with the high strain RMSE found in the models with generic material properties in the head and neck especially (Figure 2). The assigned Young’s modulus in the models with generic material properties might have been too low in these regions, leading to the observed high strains. The growth plate was not modelled with the generic approach, leading to further errors, especially in the strain calculation in the femoral head. Another problem with the models using generic material properties was the distinct boundaries between the different Young’s moduli. Especially, the strains were influenced by the neighbouring material properties. When cortical bone was modelled, the bone was stiff and shifted the strains to the elements that modelled trabecular bone again (Supplementary Figures SD,E).

There was a clear separation in stress errors between the three material modelling types. Comparing only the models with SSDM-based material properties individually per region, they reflected the same trend, which was seen in the Young’s modulus error. In both femur and tibia, higher errors in material property prediction resulted in higher stress errors. This shows the importance of accurate density predictions from the SSDM for the stress calculations. The differences in strain errors between the models with CT- and SSDM-based material properties were smaller than for stress and did not reflect the errors in the Young’s modulus, as was observed for stress. Together with the observation that models with CT-based material properties showed the lowest stress errors, this indicates that personalised material properties are more important for stresses than strains. The determination coefficients further emphasize this finding. The decrease in *R* ^2^ values was greater for stresses than strains when transitioning from CT-based to SSDM-based material properties with consistent geometry (SSDM-CT to SSDM-SSDM and Gen-CT to Gen-SSDM). In the tibia, the decrease in *R* ^2^ value is more important than in the femur, indicating that the tibia is even more sensitive to differences in material properties than the femur.

### 4.3 Biomechanical relevance

CT-derived information are usually sparse and/or missing in paediatric population. In the research context, MRI is the modality of choice for obtaining bone and muscle information in children. While patient-specific bone geometry can be extracted from MRI, bone mineral densities are not available. This study investigated whether a generic approach to determine material properties was a viable option. This study showed that using generic material properties for FEA on a paediatric population in the case of a single leg standing boundary condition is not reliable, with average R2 of 0.33 and 0.57 for stress and negative R2 for strain in the femur and tibia respectively, while having patient-specific geometry. Our SSDM bone mineral density prediction increased R2 to 0.86 and 0.80 for stress and 0.95 and 0.82 for strain in the femur and tibia when used with patient-specific geometry. When CT-based densities are not available, the SSDM prediction might be more suitable than generic material properties for a paediatric population under a single leg standing boundary condition.

Future work will investigate the effect of fitting the SSDM to the patient-specific geometry on the bone mineral density prediction accuracy and its influence on FEA results.

In summary, the differences in material properties were more pronounced. The mean stress and strain values showed clear trends related to changes in material properties. Models with generic material properties showed statistically significantly different mean values, higher RMSE and lower coefficients of determination than models with CT- or SSDM-based material properties for both stress and strain. These findings suggest that the generic material and linearly scaling geometry are insufficient to capture paediatric bone mechanical behaviour.

### 4.4 Limitations

Several limitations are associated with the current study. First, limited paediatric data is available, leading to applied boundary conditions and density-Young’s modulus equations coming from an adult dataset. The relationship between CT density, bone mineral density and Young’s modulus used in this study is based on adults (Schileo et al., 2008; Morgan et al., 2003; Keyak et al., 1994). There are several proposed relationships between density and elasticity, which are different from one another (Helgason et al., 2008). The effect of these relationships between density and elasticity is difficult to isolate and quantify. The method used to estimate bone modulus of elasticity from CT scans is reliant on findings from Öhman et al. (2011), where the author concluded that the difference in tissue strength and stiffness between paediatric and adult bone was correlated to ash density which leads to the conclusion that relationships (e.g., density–elasticity) found in adult human bones can be extended to child bone tissue. Previous studies have shown that cortical bone mean mineralisation increased until about 2 years, then remained fairly consistent (Zimmermann et al., 2019; Schileo et al., 2008).

While we have shown that ash density remains a strong predictor of stiffness across ages (Currey et al., 1996; Öhman et al., 2011), this approach primarily accounts for the mineral phase of the bone matrix. Paediatric bone is known to have a lower mineral-to-collagen ratio and different collagen cross-linking compared to mature bone. Since CT-based material mapping is essentially a proxy for mineral density, it may fail to capture the mechanical contribution of the organic matrix, which influences the post-yield behaviour and strain distribution in younger individuals. In adult FE studies, even with site-specific calibration, errors in predicted vs. experimental strains can range from 10% to 15% (52). It is conceivable that these errors are larger in paediatric models, particularly in the youngest subjects, where the bone is most compliant and least mineralised. Furthermore, as no direct experimental strain validation was performed on the specific femora and tibiae in this cohort, the reported strain values should be interpreted as comparative indices of structural competence rather than absolute physiological values.

However, we do not believe these limitations will impact the comparison made in this study. CT-based and SSDM-based elastic moduli were based on the same density-elasticity relationship, and therefore, the differences in bone mineral density between CT-based and SSDM-based models should outweigh the error derived from extrapolating the adult density-elasticity relationship to paediatric bones. Moreover, according to the recommendations from Helgason et al. (2008) the density-elasticity relationships should be from the same anatomical site on which the FE investigation is planned.

The applied loading condition (magnitude and direction) was derived from *in vivo* instrumented implants in older adults, which may not accurately represent the loading of our paediatric cohort on the hip and knee (Bergmann et al., 2010; Kutzner et al., 2010). Subject-specific paediatric joint loading datasets are currently unavailable *in vivo*, and existing paediatric musculoskeletal models rely on linear scaling of adult data. The same boundary condition definition was applied consistently across all model configurations for each participant. Consequently, any potential bias introduced by adult-derived loading directions is systematic across all modelling approaches and does not affect the comparative analyses that are the primary focus of this study. Additionally, the boundary conditions in both the femur and tibia restricted bone motion in the distal end, leading to lower strains than in the other regions. Therefore, the stress and strain errors in the distal regions could be higher for different boundary conditions. Further research will investigate the application of various boundary conditions to address this limitation. In addition, the SSDM-based models in this study were generated using regression-based estimation of principal component scores. Alternative reconstruction strategies (e.g., image-derived optimisation) may lead to different distributions of geometry and material prediction errors and could therefore influence the outcomes of the FE model and relative sensitivities reported here.

Second, the assigned generic material properties were not individually adjusted, as was done with the generic geometry. In general, Young’s modulus increases with maturation (Bavil et al., 2025; Szabo and Rimnac, 2022), and it has been shown that 25% of the BMD is accumulated during puberty, mainly in cortical bone (Choisne et al., 2025). The values used for cortical bone, trabecular bone and bone marrow were the standard found in the literature (Kainz et al., 2020). No systematic bias was found with age, meaning the error in stress and strain from the generic material properties were not influenced by age. The growth plate was not modelled with the generic material properties, leading to a discrepancy between the generic material properties and the CT- and SSDM-based material properties. Additionally, the thresholds were established based on visual inspections of the CT scans, as no data were available for paediatric lower limbs. The thresholds might not be suitable for all individuals, leading to a misclassification of the different bone types. However, the CT scans from both the lightest and the heaviest subjects in the dataset were inspected to ensure that it is appropriate to use the same thresholds for the full dataset followed by a quality assurance analysis on random 20 participants. Alternative calibration strategies could have been to use our knowledge from the CT derived bone mineral densities to create average trabecular and cortical bone mineral density by age. However, this would have been outside of the scope of this study as we wanted to use generic material properties found currently used for FEA on paediatric bones. Lastly, it is worth considering that the individual bones were not fully independent, as there were generally two limbs per subject (left and right) to increase the sample size. Also, the SSDM predicted geometry and material properties were coupled and therefore dependent on each other (Xu et al., 2025a). This becomes relevant in the context of statistical analysis, which is why we could not perform a 2-way repeated measures ANOVA (or its non-parametric equivalent) to examine the influence of geometry versus material properties. Instead, we opted for two independent Friedman tests, one with varying geometry and constant CT-based material properties and the other with varying material properties and constant CT-based geometry. Consequently, the reported relative contributions of geometry and material properties should be interpreted as approximate estimates rather than fully independent main effects, particularly for SSDM-based models where shape and density originate from a joint statistical space. Interaction effects inherent to the SSDM cannot be fully isolated with the present design and may contribute to the observed differences when SSDM-derived geometry and/or material inputs are used.

## 5 Conclusion

Our study generated FE models using CT-based, SSDM-based and generic representations of geometry and material properties to investigate the influence of geometry and material property accuracies on stresses and strains in paediatric femora and tibiae. In our dataset and under the standardized single-leg-standing load, changing material assignment from CT-based to SSDM-based to Generic led to larger changes in FE outcomes, whereas geometry differences were most evident as local errors within ROIs. Accurate material properties are more important for stress than strain calculations. The tibia appears to be more susceptible to changes in geometry and material properties than the femur, with tibial strains exhibiting the highest sensitivity. Caution should be advised when interpreting results from the SSDM-based model, as geometry and material predictions are inherently coupled in our methodology.

Further research is needed to gain a better understanding of paediatric bone material properties under various boundary conditions. The SSDM will be made available on the SimTK platform at this address https://simtk.org/projects/paed_ssm.

## Data Availability

The datasets presented in this article are not readily available because the authors do not have permission to share the dataset. Requests to access the datasets should be directed to j.choisne@auckland.ac.nz.
