# Supplementary material for: Understanding the influence of geometry and material properties on femoral and tibial stress and strain analysis in a paediatric population for single-leg standing simulation
Source: Front Bioeng Biotechnol. 2026 Jun 11;14:1752875. doi: 10.3389/fbioe.2026.1752875 (PMC13294268; doi:10.3389/fbioe.2026.1752875)
Supplement: Supplementary file 1 [file Supplementaryfile1.pdf]

Understanding the influence of geometry and material properties on femoral and tibial stress and strain analysis in a paediatric population for single-leg standing simulation – Supplementary Material

Seraina Kämpf[1,2]; Yidan Xu[2]; Julie Choisne[2,*]
1. ETH Zurich, Switzerland
2. Auckland Bioengineering Institute, The University of Auckland, Auckland, New Zealand
* j.choisne@auckland.ac.nz

Methods

Data acquisition and data extraction

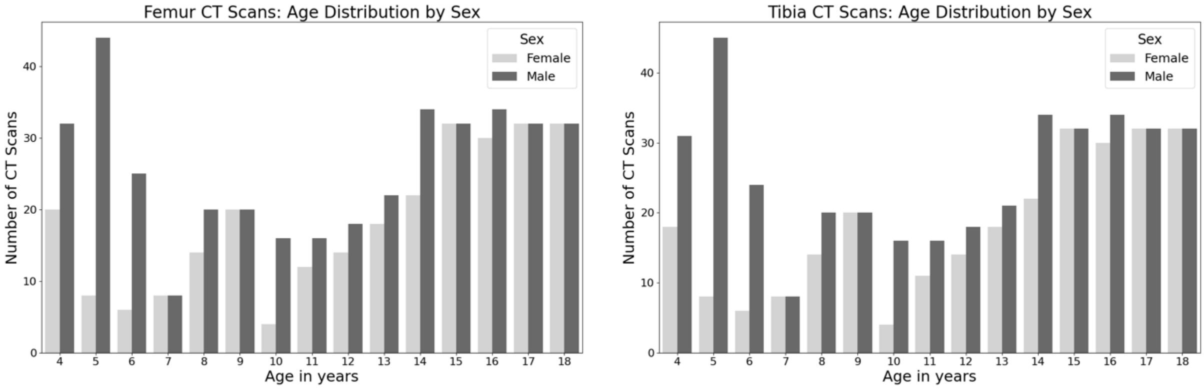

Figure A: Distribution of age and sex in the dataset of femoral (left) and tibial (right) CT scans

Table A: Number of femora and tibiae distributed by age and sex

	**Number of Femora**			**Number of Tibiae**		
**Age (years)**	Female	Male	Total	Female	Male	Total
**4**	20	32	52	18	31	49
**5**	8	44	52	8	45	53
**6**	6	25	31	6	24	30
**7**	8	8	16	8	8	16
**8**	14	20	34	14	20	34
**9**	20	20	40	20	20	40
**10**	4	16	20	4	16	20
**11**	12	16	28	11	16	27
**12**	14	18	32	14	18	32
**13**	18	22	40	18	21	39
**14**	22	34	56	22	34	56
**15**	32	32	64	32	32	64
**16**	30	34	64	30	34	64
**17**	32	32	64	32	32	64
**18**	32	32	64	32	32	64
**Total**	272	385	657	269	383	652

Table B: Average CT pixel spacing, mesh edge length and sampling ratio across the dataset. Sampling ratio = average mesh edge length / CT pixel spacing

	Age range				
	4–6 Years	7-9 Years	10-12 Years	13-15 Years	16-18 Years
Avg. CT Pixel Spacing (mm)	0.88	0.93	0.96	0.96	0.94
Avg. Mesh Edge Length (mm)	2.3	2.7	3.3	3.5	3.6
Sampling Ratio	2.6	3	3.3	3.7	3.9

Results

Mean Values

Table C1: Mean von Mises stress values (MPa) ± SD per region for each model

	Mean Stress Values ± SD (Mpa)							
	**Femoral Head**	**Femoral Neck**	**Trochanter**	**Femoral Shaft**	**Distal Femur**	**Proximal Tibia**	**Tibial Shaft**	**Distal Tibia**
**CT-CT**	2.57 ± 0.67	2.50 ± 0.64	1.48 ± 0.38	2.58 ± 0.55	0.48 ± 0.13	0.95 ± 0.22	2.90 ± 0.68	1.43 ± 0.37
**CT-SSDM**	2.58 ± 0.67	2.49 ± 0.63	1.43 ± 0.36	2.52 ± 0.54	0.48 ± 0.12	0.93 ± 0.22	2.80 ± 0.65	1.40 ± 0.36
**CT-Gen**	1.88 ± 0.44	2.48 ± 0.63	1.82 ± 0.52	3.10 ± 0.71	0.47 ± 0.12	0.90 ± 0.20	2.88 ± 0.71	1.45 ± 0.37
**SSDM-CT**	2.55 ± 0.61	2.48 ± 0.59	1.46 ± 0.34	2.54 ± 0.49	0.47 ± 0.11	0.94 ± 0.21	2.86 ± 0.57	1.42 ± 0.34
**SSDM-SSDM**	2.56 ± 0.61	2.46 ± 0.57	1.41 ± 0.32	2.47 ± 0.48	0.47 ± 0.10	0.92 ± 0.21	2.76 ± 0.56	1.39 ± 0.34
**SSDM-Gen**	1.87 ± 0.40	2.46 ± 0.58	1.79 ± 0.46	3.05 ± 0.61	0.46 ± 0.11	0.89 ± 0.19	2.82 ± 0.55	1.44 ± 0.34
**Gen-CT**	2.60 ± 0.63	2.53 ± 0.61	1.48 ± 0.37	2.58 ± 0.62	0.48 ± 0.12	0.94 ± 0.24	2.90 ± 0.72	1.44 ± 0.35
**Gen-SSDM**	2.60 ± 0.63	2.50 ± 0.60	1.43 ± 0.35	2.51 ± 0.60	0.48 ± 0.11	0.93 ± 0.23	2.81 ± 0.69	1.42 ± 0.34
**Gen-Gen**	1.90 ± 0.45	2.50 ± 0.60	1.81 ± 0.48	3.10 ± 0.74	0.47 ± 0.12	0.90 ± 0.22	2.84 ± 0.70	1.47 ± 0.36

Table C2: Mean first principal strain values (mm/m) ± SD per region for each model

	Mean Strain Values ± SD (mm/m)							
	**Femoral Head**	**Femoral Neck**	**Trochanter**	**Femoral Shaft**	**Distal Femur**	**Proximal Tibia**	**Tibial Shaft**	**Distal Tibia**
**CT-CT**	0.70 ± 0.17	0.42 ± 0.11	0.16 ± 0.04	0.12 ± 0.03	0.06 ± 0.02	0.41 ± 0.14	0.14 ± 0.04	0.29 ± 0.08
**CT-SSDM**	0.69 ± 0.16	0.41 ± 0.10	0.16 ± 0.03	0.12 ± 0.02	0.06 ± 0.01	0.41 ± 0.10	0.15 ± 0.03	0.29 ± 0.06
**CT-Gen**	2.03 ± 0.48	1.27 ± 0.28	0.40 ± 0.10	0.09 ± 0.02	0.15 ± 0.03	0.51 ± 0.11	0.08 ± 0.02	0.49 ± 0.10
**SSDM-CT**	0.70 ± 0.17	0.42 ± 0.11	0.16 ± 0.04	0.12 ± 0.03	0.06 ± 0.01	0.41 ± 0.11	0.14 ± 0.04	0.29 ± 0.08
**SSDM-SSDM**	0.69 ± 0.14	0.40 ± 0.08	0.15 ± 0.03	0.11 ± 0.02	0.06 ± 0.01	0.41 ± 0.08	0.15 ± 0.03	0.28 ± 0.05
**SSDM-Gen**	2.02 ± 0.43	1.26 ± 0.24	0.40 ± 0.06	0.09 ± 0.02	0.15 ± 0.03	0.50 ± 0.10	0.08 ± 0.01	0.49 ± 0.09
**Gen-CT**	0.72 ± 0.19	0.42 ± 0.12	0.16 ± 0.04	0.12 ± 0.03	0.06 ± 0.02	0.41 ± 0.12	0.14 ± 0.05	0.30 ± 0.11
**Gen-SSDM**	0.70 ± 0.15	0.41 ± 0.09	0.16 ± 0.04	0.012 ± 0.03	0.06 ± 0.01	0.40 ± 0.10	0.15 ± 0.04	0.29 ± 0.07
**Gen-Gen**	2.06 ± 0.49	1.30 ± 0.33	0.40 ± 0.10	0.09 ± 0.02	0.15 ± 0.04	0.50 ± 0.12	0.08 ± 0.02	0.51 ± 0.16

Statistical Analysis

Friedman tests

Table D: Statistics from Friedman tests (χ^2- and p-values) for each region of interest in the femur and tibia. P-values < 0.05 were considered statistically significant.

	CT-CT vs. CT-SSDM vs CT-Gen		CT-CT vs. SSDM-CT vs. Gen-CT	
	Von Mises stress	1st principal strain	Von Mises stress	1st principal strain
Femur	$\chi^2(2) = 1096.924$, $p < 0.001$	$\chi^2(2) = 987.735$, $p < 0.001$	$\chi^2(2) = 11.588$, $p = 0.003$	$\chi^2(2) = 8.802$, $p = 0.012$
Femoral Head	$\chi^2(2) = 990.250$, $p < 0.001$	$\chi^2(2) = 1000.151$, $p < 0.001$	$\chi^2(2) = 1.062$, $p = 0.588$	$\chi^2(2) = 1.961$, $p = 0.375$
Femoral Neck	$\chi^2(2) = 41.930$, $p < 0.001$	$\chi^2(2) = 1013.631$, $p < 0.001$	$\chi^2(2) = 8.533$, $p = 0.014$	$\chi^2(2) = 7.269$, $p = 0.026$
Trochanter	$\chi^2(2) = 1068.578$, $p < 0.001$	$\chi^2(2) = 1041.288$, $p < 0.001$	$\chi^2(2) = 9.957$, $p = 0.007$	$\chi^2(2) = 4.560$, $p = 0.102$
Femoral Shaft	$\chi^2(2) = 1140.749$, $p < 0.001$	$\chi^2(2) = 1008.508$, $p < 0.001$	$\chi^2(2) = 12.228$, $p = 0.002$	$\chi^2(2) = 10.704$, $p = 0.005$
Distal Femur	$\chi^2(2) = 413.927$, $p < 0.001$	$\chi^2(2) = 1100.353$, $p < 0.001$	$\chi^2(2) = 5.610$, $p = 0.060$	$\chi^2(2) = 7.004$, $p = 0.030$
Tibia	$\chi^2(2) = 513.095$, $p < 0.001$	$\chi^2(2) = 774.617$, $p < 0.001$	$\chi^2(2) = 1.610$, $p = 0.447$	$\chi^2(2) = 1.144$, $p = 0.564$
Proximal Tibia	$\chi^2(2) = 815.929$, $p < 0.001$	$\chi^2(2) = 787.840$, $p < 0.001$	$\chi^2(2) = 0.518$, $p = 0.772$	$\chi^2(2) = 7.090$, $p = 0.029$
Tibial Shaft	$\chi^2(2) = 485.199$, $p < 0.001$	$\chi^2(2) = 1058.014$, $p < 0.001$	$\chi^2(2) = 1.558$, $p = 0.459$	$\chi^2(2) = 4.264$, $p = 0.119$
Distal Tibia	$\chi^2(2) = 599.543$, $p < 0.001$	$\chi^2(2) = 982.266$, $p < 0.001$	$\chi^2(2) = 0.307$, $p = 0.858$	$\chi^2(2) = 3.854$, $p = 0.146$

Wilcoxon Signed-rank tests

Table E: Z-scores and p-values from post-hoc analysis with Wilcoxon signed-rank test for mean von Mises stress and mean first principal strain in the femur, tibia and their corresponding ROIs. A Bonferroni correction was applied, resulting in a new significance level $p_{new} = 0.017$. If the Friedman test showed no significant difference ($p \geq 0.05$), no post-hoc analysis was performed and the corresponding sections in the table show N/A.

**Von Mises stress**	CT-CT vs. CT-SSDM	CT-CT vs. CT-Gen	CT-SSDM vs. CT-Gen	CT-CT vs. SSDM-CT	CT-CT vs. Gen-CT	SSDM-CT vs. Gen-CT
Femur	Z = -16.845, p < 0.001	Z = -22.159, p < 0.001	Z = -22.190, p < 0.001	Z = -3.622, p < 0.001	Z = -1.235, p = 0.217	Z = -0.979, p = 0.328
Femoral Head	Z = -4.343, p < 0.001	Z = -22.206, p < 0.001	Z = -22.206, p < 0.001	N/A	N/A	N/A
Femoral Neck	Z = -6.317, p < 0.001	Z = -6.273, p < 0.001	Z = -1.953, p = 0.051	Z = -1.943, p = 0.52	Z = -0.55, p = 0.956	Z = -0.683, p = 0.495
Trochanter	Z = -16.617, p < 0.001	Z = -22.162, p < 0.001	Z = -22.192, p < 0.001	Z = -3.189, p = 0.001	Z = -0.978, p = 0.328	Z = -0.629, p = 0.529
Femoral Shaft	Z = -18.348, p < 0.001	Z = -22.206, p < 0.001	Z = -22.206, p < 0.001	Z = -3.275, p = 0.001	Z = -1.373, p = 0.170	Z = -1.275, p = 0.202
Distal Femur	Z = -0.178, p = 0.859	Z = -15.265, p < 0.001	Z = -18.268, p < 0.001	N/A	N/A	N/A
Tibia	Z = -20.229, p < 0.001	Z = -11.907, p < 0.001	Z = -13.384, p < 0.001	N/A	N/A	N/A
Proximal Tibia	Z = -14.403, p < 0.001	Z = -20.810, p < 0.001	Z = -21.488, p < 0.001	N/A	N/A	N/A
Tibial Shaft	Z = -20.996, p < 0.001	Z = -7.440, p < 0.001	Z = -14.075, p < 0.001	N/A	N/A	N/A
Distal Tibia	Z = -14.019, p < 0.001	Z = -8.994, p < 0.001	Z = -21.600, p < 0.001	N/A	N/A	N/A

**1st principal strain**	CT-CT vs. CT-SSDM	CT-CT vs. CT-Gen	CT-SSDM vs. CT-Gen	CT-CT vs. SSDM-CT	CT-CT vs. Gen-CT	SSDM-CT vs. Gen-CT
Femur	Z = -2.297, p = 0.022	Z = -22.190, p < 0.001	Z = -22.190, p < 0.001	Z = -2.612, p = 0.009	Z = -0.067, p = 0.947	Z = -0.871, p = 0.384
Femoral Head	Z = -2.538, p = 0.011	Z = -22.207, p < 0.001	Z = -22.207, p < 0.001	N/A	N/A	N/A
Femoral Neck	Z = -3.640, p < 0.001	Z = -22.207, p < 0.001	Z = -22.208, p < 0.001	Z = -1.240, p = 0.215	Z = -0.674, p = 0.500	Z = -0.738, p = 0.460
Trochanter	Z = -6.097, p < 0.001	Z = -22.193, p < 0.001	Z = -22.196, p < 0.001	N/A	N/A	N/A
Femoral Shaft	Z = -2.334, p = 0.020	Z = -21.739, p < 0.001	Z = -22.528, p < 0.001	Z = -3.099, p = 0.002	Z = -0.238, p = 0.812	Z = -2.407, p = 0.016
Distal Femur	Z = -0.030, p = 0.976	Z = -22.249, p < 0.001	Z = -22.263, p < 0.001	Z = -3.481, p < 0.001	Z = -0.202, p = 0.840	Z = -3.222, p = 0.001
Tibia	Z = -2.318, p = 0.020	Z = -19.026, p < 0.001	Z = -22.122, p < 0.001	N/A	N/A	N/A
Proximal Tibia	Z = -1.302, p = 0.193	Z = -18.822, p < 0.001	Z = -22.172, p < 0.001	Z = -0.608, p = 0.543	Z = -1.032, p = 0.302	Z = -2.503, p = 0.012
Tibial Shaft	Z = -9.197, p < 0.001	Z = -22.152, p < 0.001	Z = -22.278, p < 0.001	N/A	N/A	N/A
Distal Tibia	Z = -0.615, p = 0.539	Z = -22.081, p < 0.001	Z = -22.137, p < 0.001	N/A	N/A	N/A

Root Mean Square Error

Von Mises Stress RMSE

1)

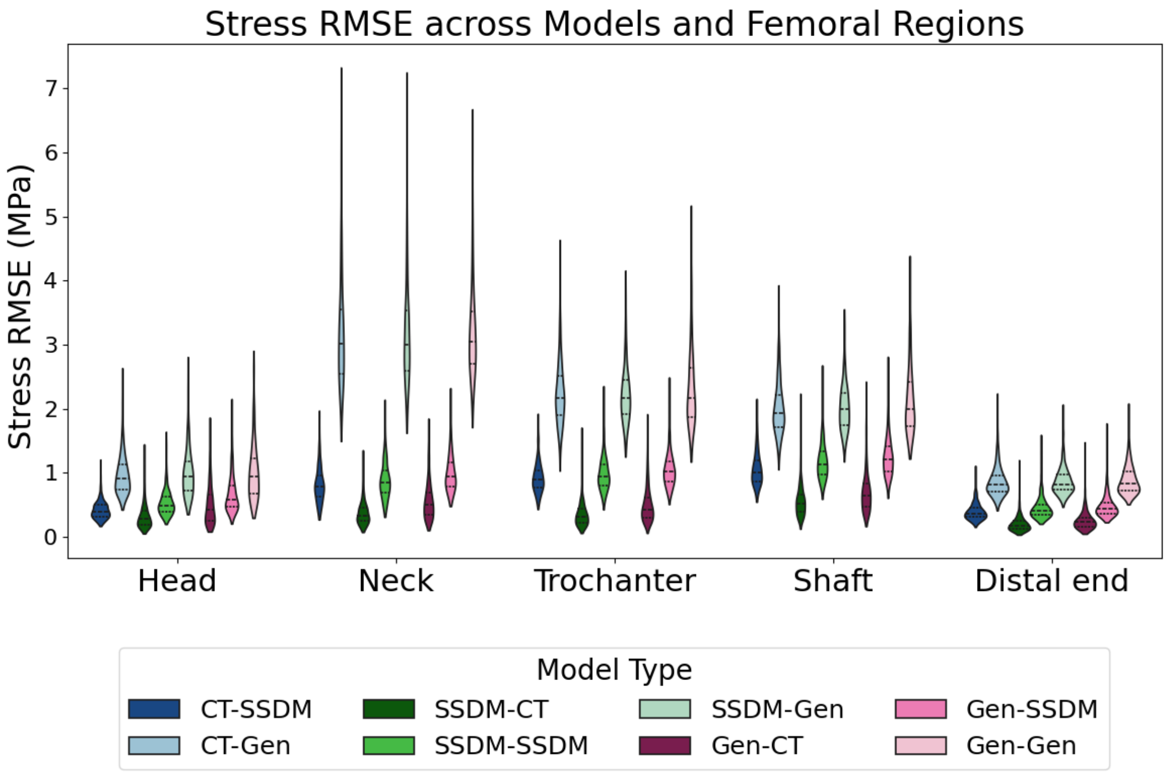

2)

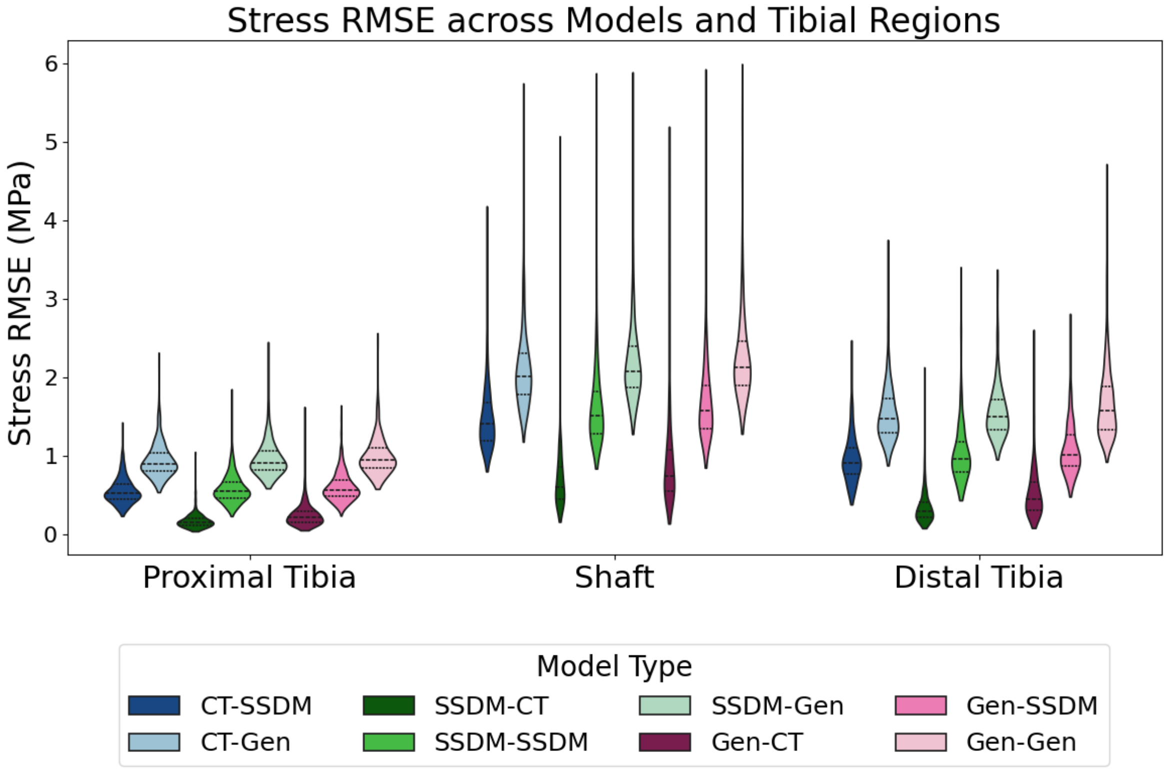

Figure B: Von Mises Stress RMSE for each model across (1) femoral and (2) tibial regions of interest compared to the subject-specific geometry and material properties (CT-CT) using violin plots.

First Principal Strain RMSE

1)

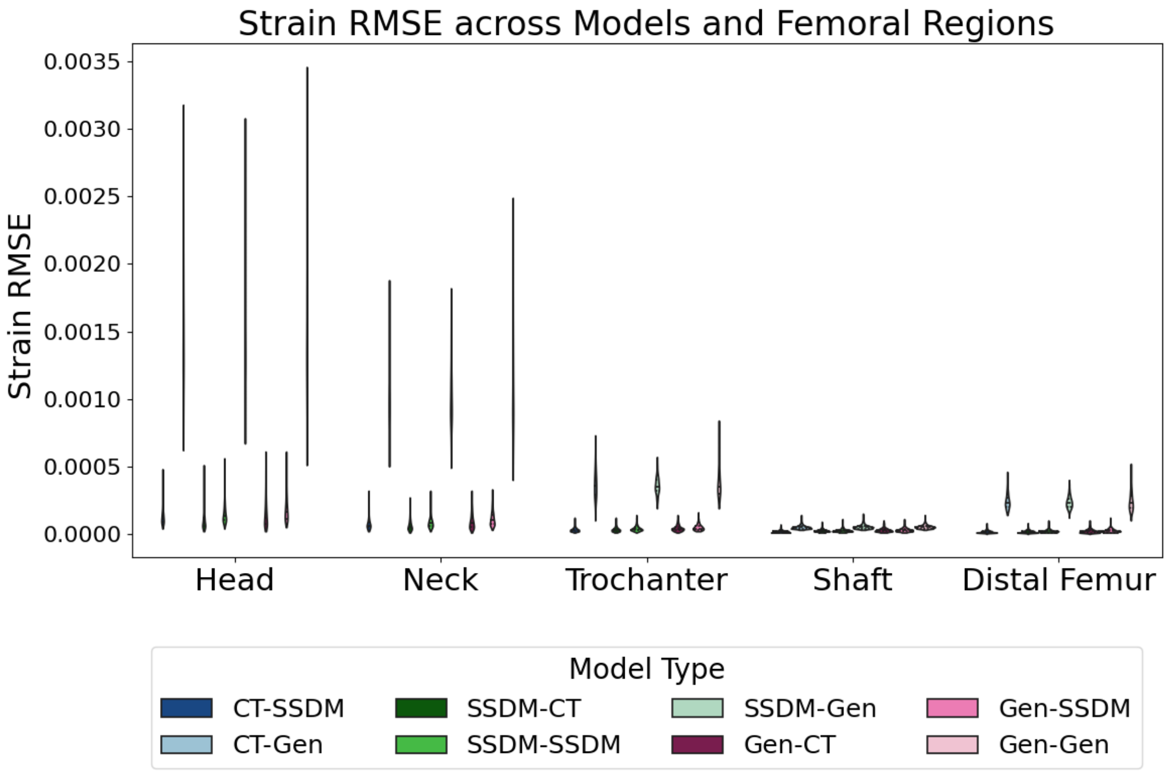

2)

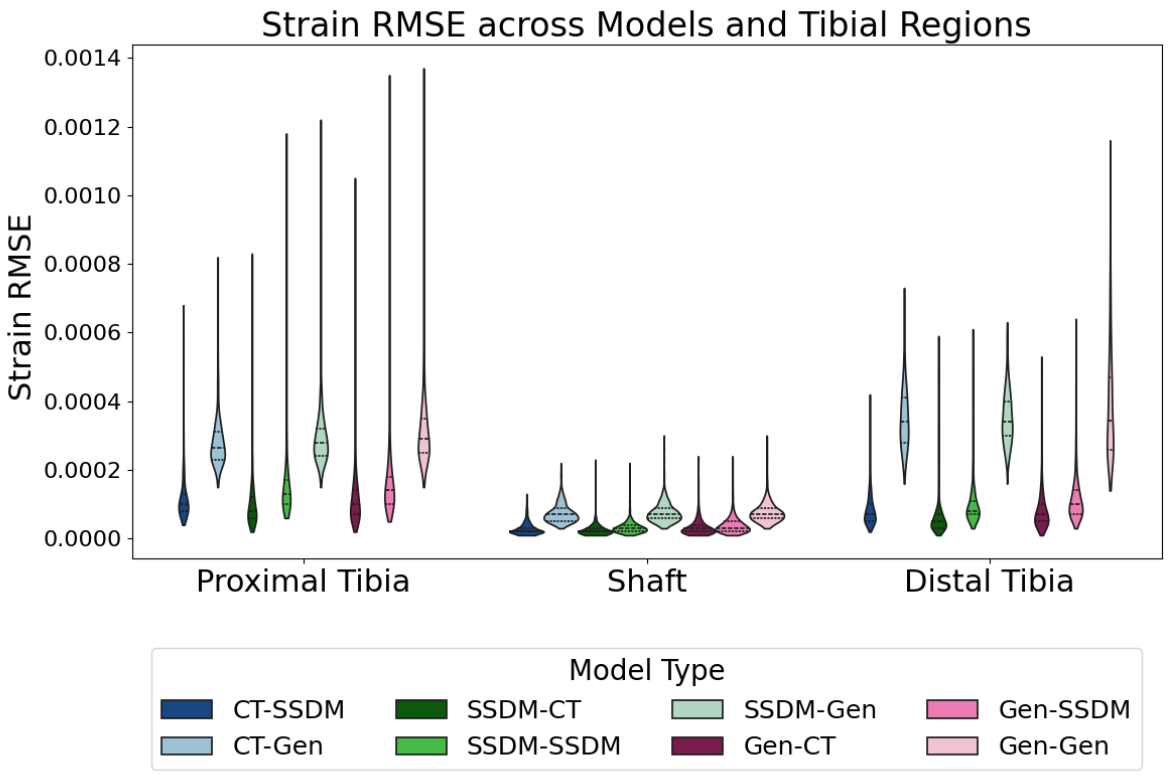

Figure C: First principal strain RMSE for each model across (1) femoral and (2) tibial regions of interest compared to the subject-specific geometry and material properties (CT-CT) using violin plots.

Coefficient Determination

First Principal Strain

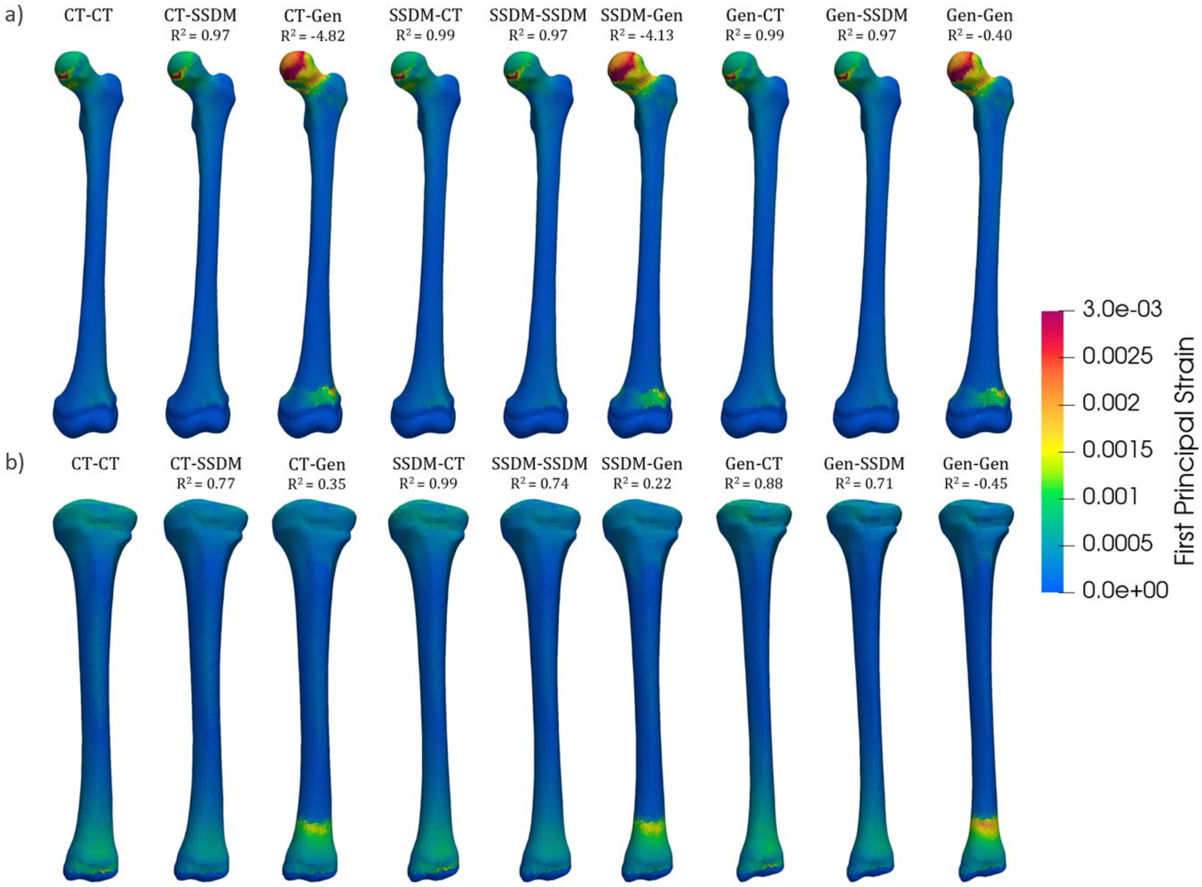

Figure D: Femoral (a) and tibial (b) models with corresponding determination coefficients (R2) for the participants with the highest first principal strain R2 score from the respective dataset. Participant (a): F, 17 years, H: 162 cm, W: 53 kg, highest score in the SSDM-CT and Gen-CT model (0.99); Participant (b): F, 5 years, H: 111 cm, W: 19 kg, highest score in the SSDM-CT model (0.99).

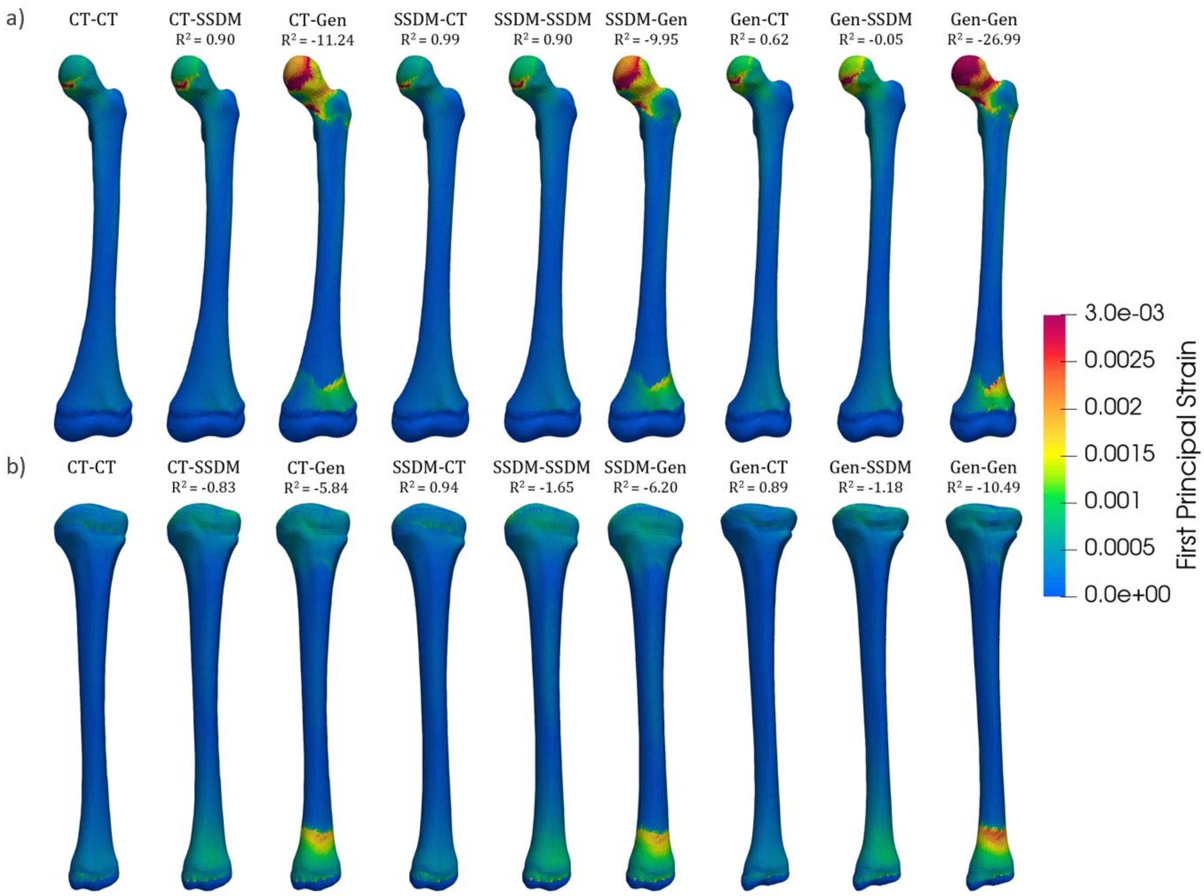

Figure E: Femoral (a) and tibial (b) models with corresponding determination coefficients (R2) for the participants with the lowest first principal strain R2 score from the respective dataset. Participant (a): M, 5 years, H: 117 cm, W: 28 kg, lowest score in the Gen-Gem model (-26.99); Participant (b): F, 4 years, 97 cm, 16 kg, lowest score in the Gen-Gen model (-10.49).

Segmentation

Table F: CT scan screen shots at the hip, knee and ankle from the frontal and sagittal view overlaid with the segmentation in red (after wrapping and smoothing) for different age ranges.

		**Hip**	**Knee**	**Ankle**
**4-6 years**	**Frontal view**	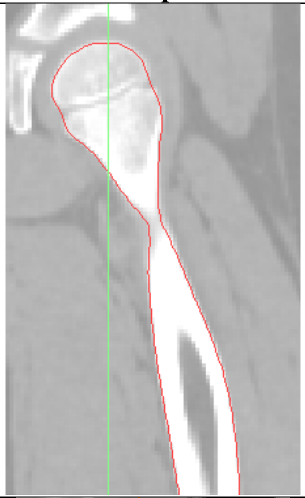	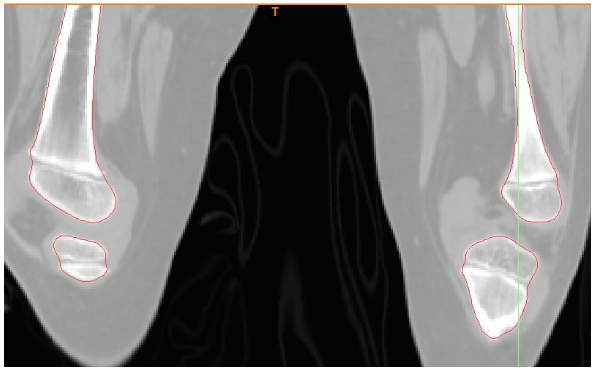	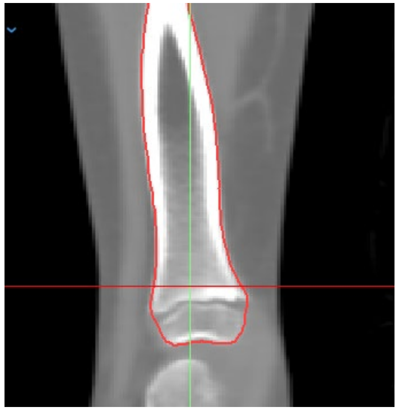
	**Sagittal view**	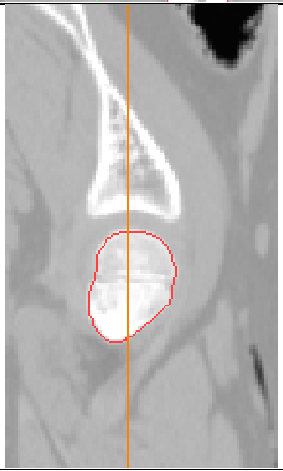	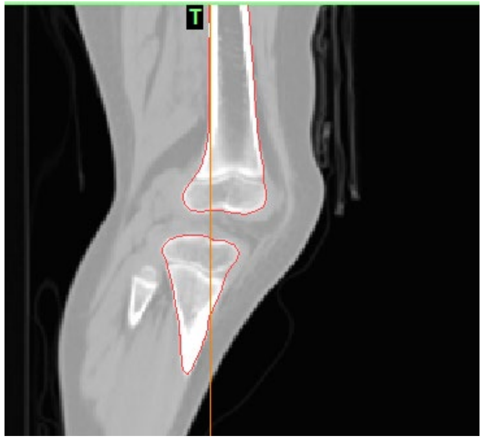	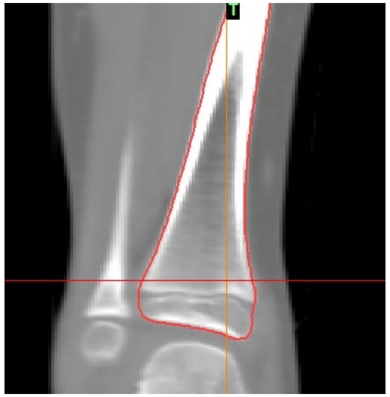

7-9 years	Frontal view	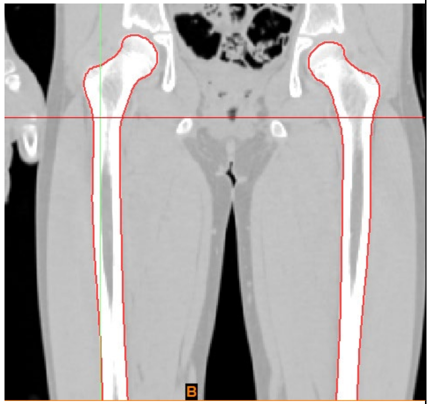	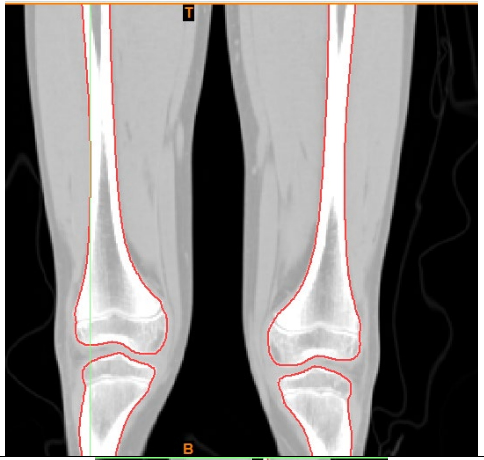	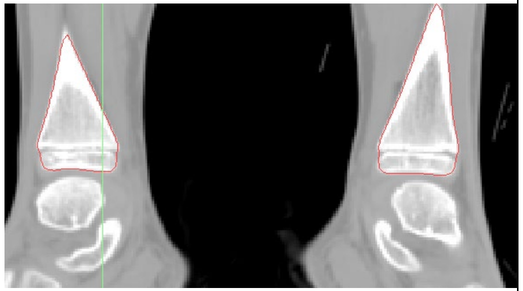
	Sagittal view	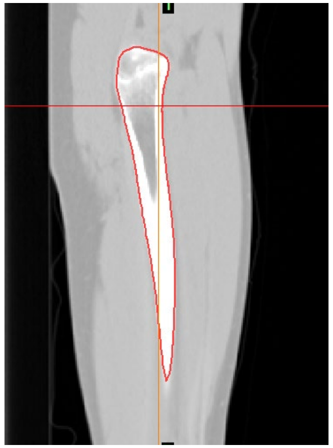	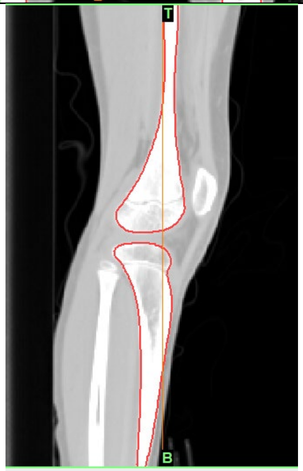	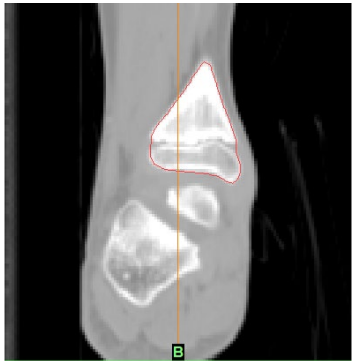

10-12 years	**Frontal view**	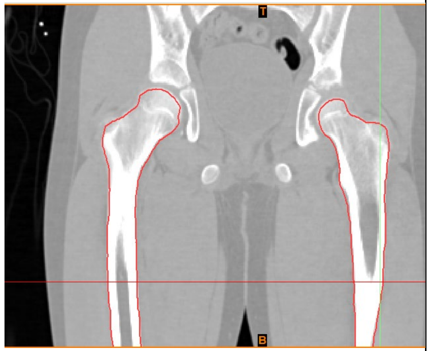	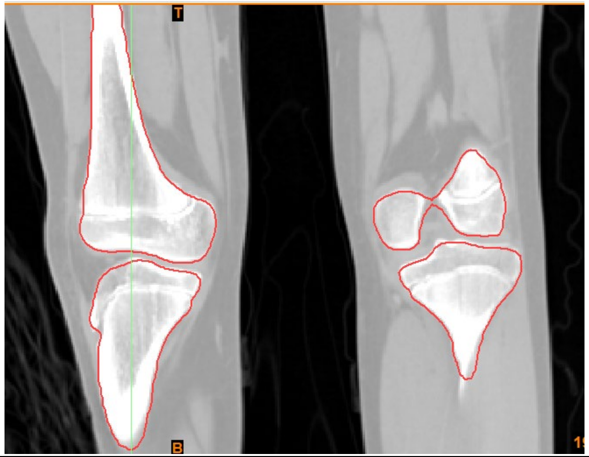	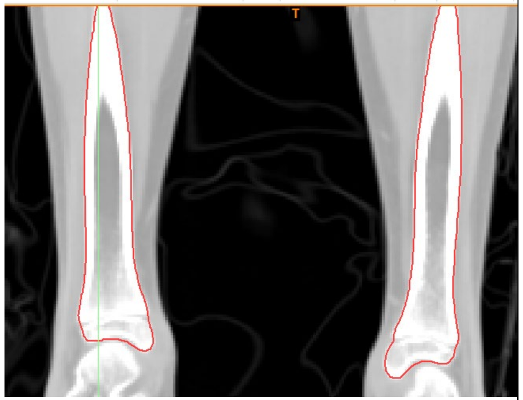
	**Sagittal view**	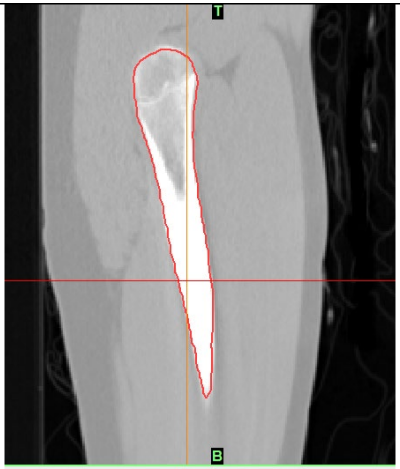	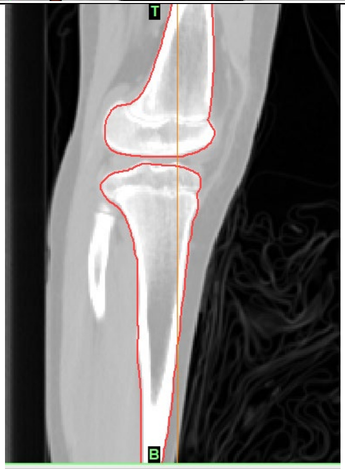	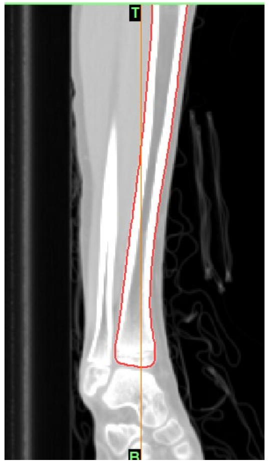

13-15 years	Frontal view	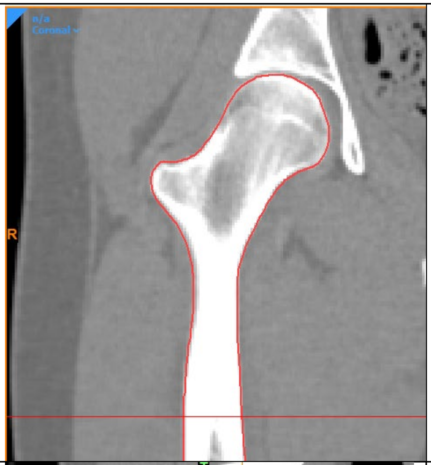	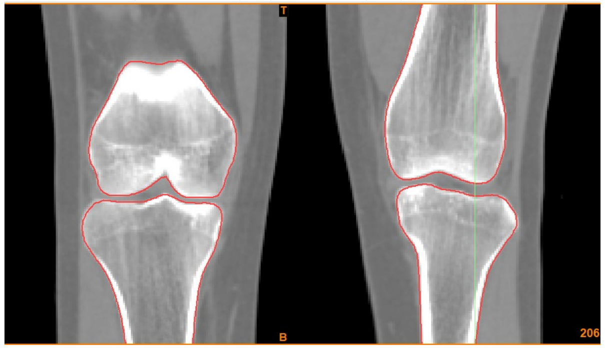	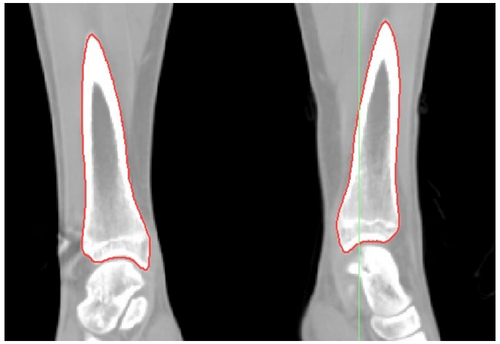
	Sagittal view	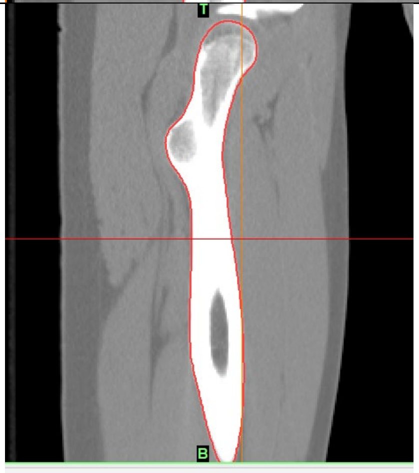	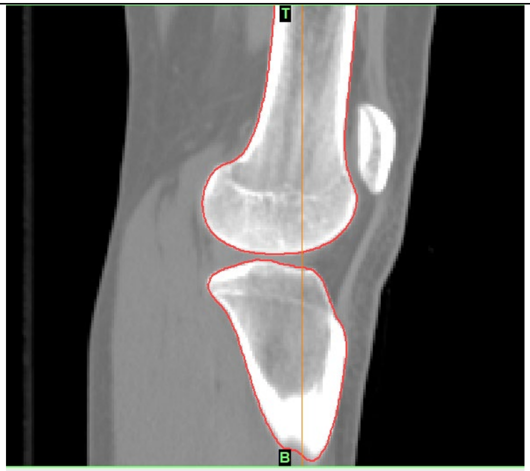	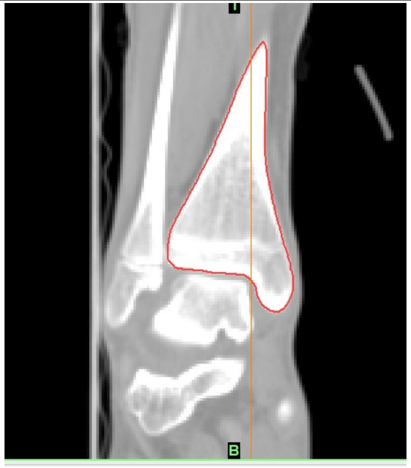

**16-18 years**	**Frontal view**	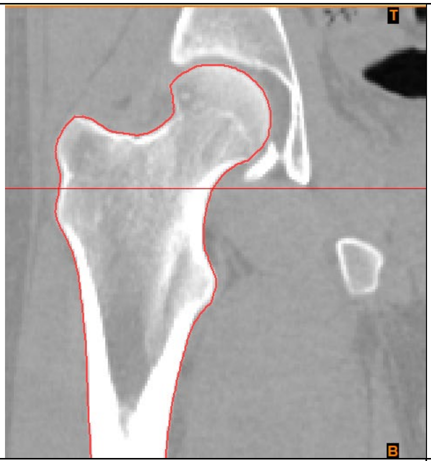	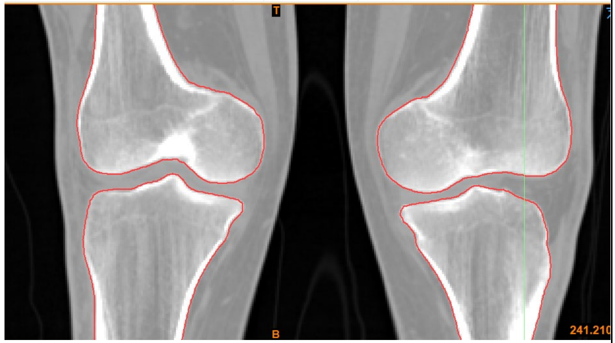	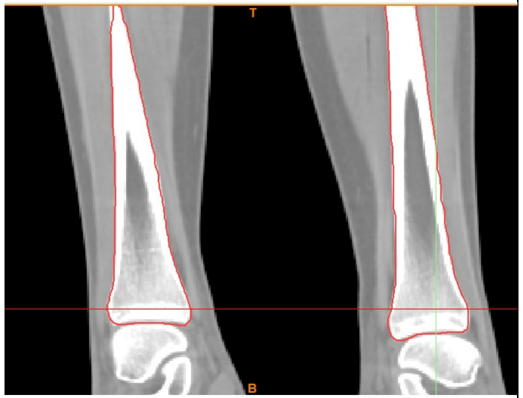
	**Sagittal view**	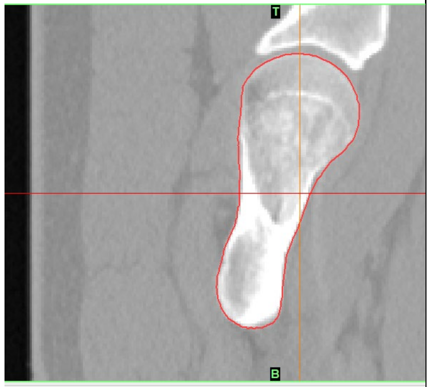	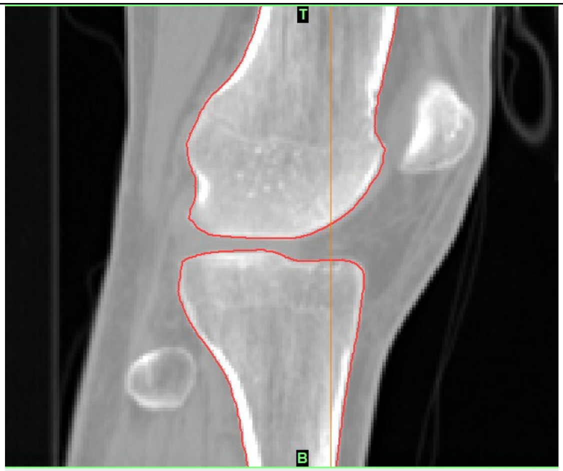	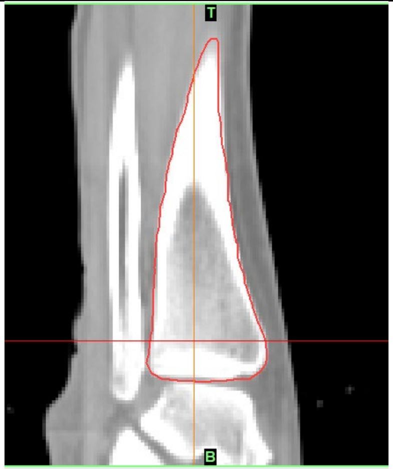
